# Pyoderma Gangrenosum: A Review of Clinical Features and Outcomes of 23 Cases Requiring Inpatient Management

**DOI:** 10.1155/2014/461467

**Published:** 2014-10-08

**Authors:** Mingwei Joel Ye, Joshua Mingsheng Ye

**Affiliations:** ^1^Department of Dermatology, Western Hospital, Footscray, VIC 3011, Australia; ^2^Department of Medicine, Dentistry and Health Sciences, University of Melbourne, Parkville, VIC 3010, Australia

## Abstract

Pyoderma gangrenosum (PG) is a rare dermatological disorder characterised by the rapid progression of a painful, necrolytic ulcer. This study retrospectively identified patients who were admitted and treated for PG during a 10-year period (2003–2013). Twenty-three patients were included in this study, 16 women and seven men. The mean age at initial admission was 62.8 years (range 30 to 89 years). Lesions were localised to lower limb in 13 patients, peristomal region in four, breast in three, and upper limb in one, and two patients had PG at multiple sites. The variants of PG noted were ulcerative (18), bullous (2), vegetative (2), and pustular (1). Associated systemic diseases were observed in 11 patients (47.8%). Systemic therapies were initiated in 21 patients while two patients received topical treatments. The mean length of hospital stay was 47 days (range 5 to 243 days) and five patients died during their admissions. Seven patients required readmissions for exacerbations of their PG. Our study showed that patients admitted for treatment of PG had high morbidity and mortality. This study also highlights the importance of early and aggressive treatment of patients admitted with PG as well as treating associated systemic diseases and wound infections.

## 1. Introduction

Pyoderma gangrenosum (PG) is a rare dermatological condition that was first described by Brocq, a French dermatologist, in 1916 [1]. It is characterized by rapidly, progressing ulceration of the skin with an ill-defined border and can occur at any age, but more frequently observed in adults than children [2]. It has a gender predilection for females [3] and commonly affects the lower extremities, in particular the pretibial area [2, 3].

The etiology of PG remains unknown but has been attributed to reactive neutrophilic dermatosis. Pathergy, a term used to describe an exaggerated skin injury occurring after trauma, can exacerbate PG [2]. Diagnosis of PG requires clinicopathologic correlation and is often a diagnosis of exclusion after common causes of skin ulceration such as infection, malignant neoplasms, and vasculitic syndromes have been ruled out. Histopathological findings of PG are not specific. Early lesions may reveal dermal neutrophilia centered on follicles, while severe skin lesions may show tissue necrosis with surrounding mononuclear cell infiltrates [2]. PG is often associated with systemic diseases such as inflammatory bowel disease (IBD), rheumatoid, and haematological conditions [4–6].

Systemic therapy such as corticosteroids and cytotoxic agents are the treatment of choice for rapidly progressing PG [7, 8]. Newer biological agents such as infliximab and adalimumab have also been found to be effective [9, 10]. Despite advances in medical therapy, the prognosis of PG remains unpredictable and, if left untreated, almost always fatal. This retrospective study was undertaken to strengthen current knowledge and experience of the outcomes of PG, as well as identifying possible factors that may exert influence over patients' outcomes.

## 2. Methods

In this study, we retrospectively analysed the characteristics of patients who were treated for PG. Twenty-three patients who were admitted and treated for PG were identified from Western Hospital Health Information Service through a search of medical records over a 10-year period from July 2003 to September 2013.

The medical records of these patients were reviewed and the following data were extracted: age at initial hospital admission for PG, sex, clinical variant of PG, site of ulcer, associate systemic diseases, investigation results, treatment regimes, and outcomes including length of hospital stay, deaths, and recurrence during follow-up.

## 3. Results

### 3.1. Patient Demographics

Twenty-three patients (see Table 1) were included in this study between July 2003 and September 2013. All patients were admitted for inpatient management of PG. One patient also suffered from community acquired pneumonia at the time of admission. There were 16 women and seven men (ratio of 2.3 : 1) and the mean age of onset was 62.8 years (range 30 to 89 years). The mean age of onset was 63.6 years for women and 61 years for men. The peak incidence of onset of PG was in the seventh decade (*n* = 6, 26%).

### 3.2. Clinical Features

**Figure 1:**
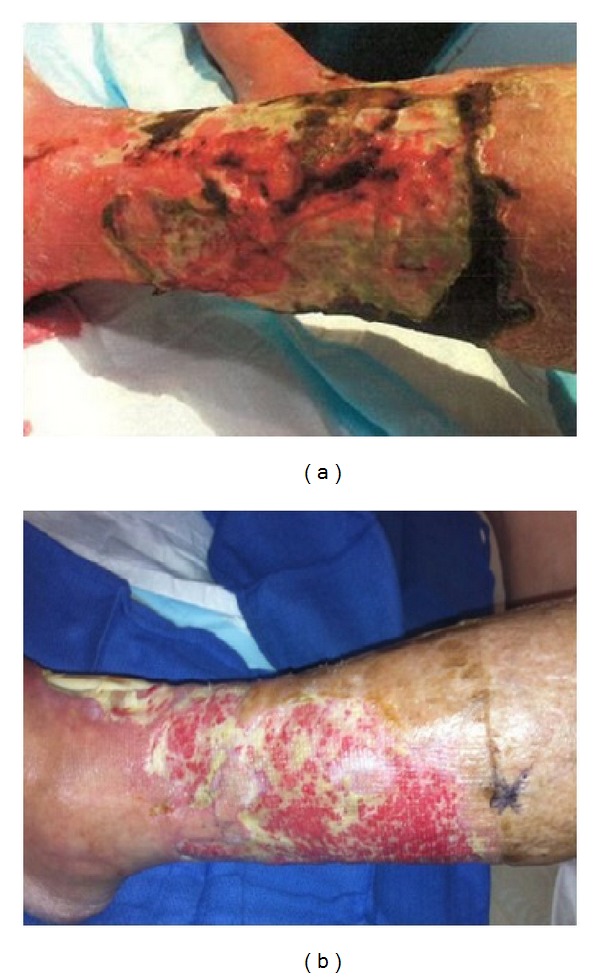
(a) 73-year-old female with bilateral lower limb ulcerative PG and colorectal cancer. (b) Epithelialization and granulation tissue formation after 3 months of prednisolone 30 mg/daily and mycophenolate mofetil 1 g/twice daily.

Ulcerative PG was the most common variant and was observed in 18 patients (78.3%), vegetative PG in two (8.7%), and bullous PG in two (8.7%) and one patient suffered from pustular PG (4.3%). The lower limb was the commonest site of PG occurrence (*n* = 13, 56.5%) (see Figure 1). For the rest of the patients, lesions developed on the breasts in three patients (13%), peristomal area in four patients (17.4%), and upper limb in one patient (4.4%) and two patients had lesions in multiple sites (8.7%). Ten patients reported trauma as a precipitating cause of PG. Of these, surgery accounted for six cases.

Other associated systemic diseases were found in 11 patients (47.8%), five cases with solid tumours (three bowel and two lung cancers), two with IBD (Crohn's disease and ulcerative colitis), two with connective tissue joint diseases (CREST and ankylosing spondylitis), and two with haematological disorders (essential thrombocythemia and monoclonal gammopathy).

### 3.3. Investigations

Wound swabs and C-reactive protein (CRP) were performed on admission. Microbiological study of swabs from the ulcers revealed positive cultures in 13 patients (56.5%).* Staphylococcus aureus* was found in five,* Enterococci* in one,* Escherichia coli* in two,* Streptococci* in four,* Pseudomonas aeruginosa* in three, and* Serratia marcescens* in one. The CRP values of our patients ranged from 3 mg/L to 474 mg/L (normal reference interval, 0–10 mg/L).

In order to exclude other causes of skin ulceration and identify underlying systemic diseases, the patients also underwent a range of laboratory tests including a full blood examination, serum electrolytes, immunoelectrophoresis, antinuclear antibodies, and rheumatoid factor. Additional tests such as hepatitis serology, antineutrophilic cytoplasmic antibodies, and extractible nuclear antigen were performed in some patients according to clinical suspicion and initial investigation results. None of our patients had a positive vasculitis test. One patient had oligoclonal banding in gamma region on immunoelectrophoresis and was subsequently diagnosed with monoclonal gammopathy.

The results of skin biopsies were available for 15 patients. Neutrophil infiltration to deep dermis was seen in 12 cases, lymphocytic infiltrate in six, abscess formation in three, vasculitis in one, and leukocytoclasia in one.

### 3.4. Treatment

**Figure 2:**
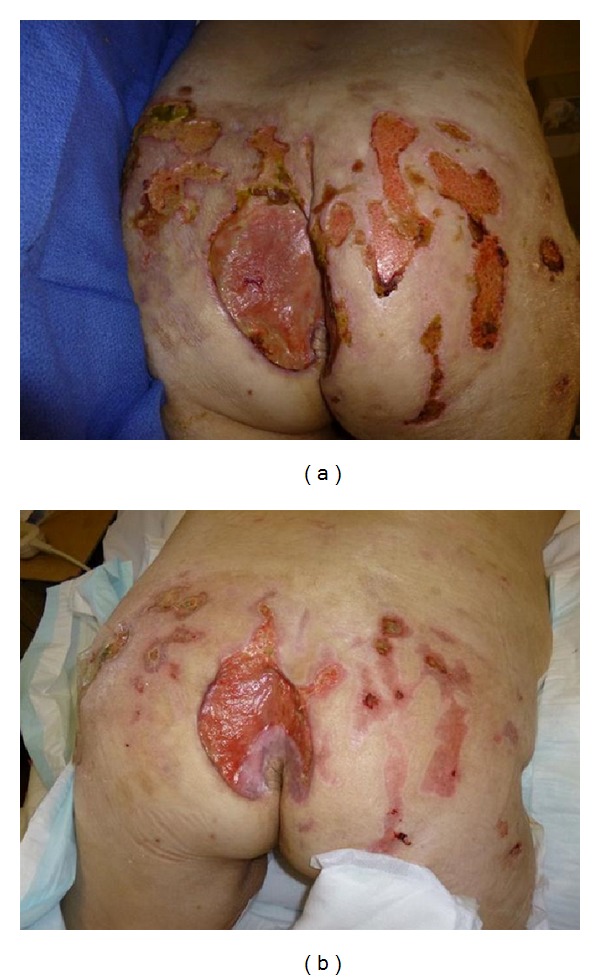
(a) 51-year-old female with PG at multiple sites including sacrum. (b) Resolution of smaller lesions after 1 month of prednisolone 50 mg/daily and mycophenolate mofetil 1 g/twice daily.

Systemic therapy was used in 21 patients (91.3%). Systemic therapy involved either monotherapy with prednisolone (dose of 25 mg–50 mg daily) in eight patients or combination therapy (see Figure 2) which consisted of at least prednisolone and other treatments such as tetracycline, dapsone, azathioprine, mycophenolate mofetil (MMF), and adalimumab. Potent topical steroids such as betamethasone dipropionate and mometasone furoate were used in two patients (8.7%).

Seven patients underwent surgical interventions while on immunosuppressive therapy. Five patients had minor debridement of their wounds, one patient had split skin grafts (SSG), and another had both SSG and minor debridement. None of the patients had exacerbation after these procedures.

Intravenous antibiotics were administered to 18 patients based on clinical suspicion of infected PG wound.

### 3.5. Patient Outcomes

#### 3.5.1. Length of Stay (LOS)

Mean length of hospital stay (LOS) till discharge or death was 47 days (range 5 to 243 days). Three patients were hospitalised for more than three months. All three suffered from concomitant wound infections during their hospital stay. Two of them grew* Staphylococcus aureus* and one grew Extended-spectrum beta-lactamase (ESBL)* Escherichia coli*. All three patients had abnormally high CRP levels (range 53 mg/L–178 mg/L) and one patient who also suffered from colorectal cancer died during the initial hospital admission.

#### 3.5.2. Death

Five patients (21.7%) died during their initial hospital stay (see Table 2). Their mean age at time of admission was 78.8 years (range 72 to 85 years). The mean LOS till death was 50.6 days (range 15 to 151 days). All had lower limb PG. The cause of death was sepsis in four patients while one patient died of multiorgan failure secondary to hypovolemia. Four patients had ulcerative and one had vegetative PG. All patients had positive wound cultures and all had highly elevated CRP levels above 50 mg/L. Four patients had associated systemic disease. All underwent systemic therapy: three had combination therapy while two had monotherapy.

#### 3.5.3. Recurrence

Seven patients had recurrence of PG requiring readmission (see Table 3). The mean number of readmissions for PG was three (range 1 to 7). Three patients died during their subsequent admissions. The mean age at diagnosis of the patients who died during subsequent admissions was 68 years. Of those who died, two patients had concomitant gastrointestinal disorders.

## 4. Discussion

### 4.1. Patient Demographics

We retrospectively reviewed 23 patients diagnosed with PG and treated at our hospital over a 10-year period. The mean age of onset was 62.8 years which was similar to that reported in two recent studies [11, 12]. Three earlier studies reported an earlier mean age of onset with peak incidence in the fifth to sixth decade [13–15]. It is widely known that PG has a predilection for females [3] and our study has shown a similar result.

### 4.2. Clinical Features

Four variants have been described in the literature, namely, ulcerative [2], vegetative [16], bullous [6], and pustular [17]. Ulcerative PG, which is the classical variant, is the commonest form in our study as well as previous studies [11–15, 18]. It is characterised by the appearance of a painful, irregular ulcer with a violaceous border [2]. The other variants of PG are less common and usually respond well to immunosuppressive treatments [3]. In our study, the lesions were more commonly localised to the lower limbs (56.5%) but this proportion was lower than other studies which reported a proportion of 70% to 80% [3, 12, 13, 15]. Pathergy was a precipitating factor in almost half the cases. One of the main causes of pathergy was surgery. It is therefore critical for clinicians to be aware and vigilant in diagnosing this complication as delayed diagnosis can potentially lead to poorer prognosis.

Many studies have reported that, in 50 to 70% of the cases, PG is associated with an underlying disease such as IBD, inflammatory arthritis, haematological disorders, and solid malignancies [11–15]. In our study, 47.8% of patients had associated systemic diseases. Most patients presented with known systemic disorders. Only one patient was newly diagnosed with an associated systemic disease during the acute admission. He was diagnosed with monoclonal gammopathy on serum protein electrophoresis. This highlights the importance of screening patients with PG for associated systemic diseases.

### 4.3. Investigations

It is not uncommon for PG to occur with wound infection. We observed that CRP alone is not specific for wound infection [6, 19, 20]. An elevated CRP can indicate either a concomitant bacterial infection or active inflammatory process associated with PG. However, an abnormally high level of CRP more than 50 mg/L, a positive wound culture, and clinical signs such as erythema and swelling indicate a wound infection which should prompt treatment with antibiotics. Immunosuppression should still be continued to prevent progression of PG [6, 19, 20] except in the presence of systemic sepsis. CRP is a valuable investigation and has been shown in previous studies to be useful in monitoring progression of PG [11].

Histology findings are nonspecific but can serve to exclude infection, malignancy, and vasculitis [2]. Neutrophilic infiltration into dermis is the histological hallmark of PG [2] and is consistent with the results of our study. Other histological findings of leukocytoclasia, abscess, and vasculitis are also seen in our patients.

### 4.4. Treatment

Systemic therapy is the mainstay of treatment for severe, progressive PG which is commonly seen in patients requiring hospital treatment for PG [7, 8]. Twenty-one patients received systemic therapy. Only two patients with mild PG received topical therapy and were discharged after a relatively short hospital stay. One patient was admitted as she was also suffering from community acquired pneumonia while the other had peristomal PG and required admission to monitor his underlying Crohn's disease.

Systemic corticosteroids have been shown to be effective in a number of studies and are therefore considered as first-line therapy [7, 8]. Combination therapy of corticosteroid and other immunosuppressive agents can be used to avoid higher doses of steroids and thus reduce the side effects associated with high doses of steroids [8]. Recently, it has been shown that antitumour necrosis factor drugs such as infliximab and adalimumab are successful in treating PG associated with IBD [9, 10]. In our study, only 1 patient received such therapy, 33-year-old female patient who was on multiple systemic immunosuppressive agents including prednisolone, MMF, azathioprine, and adalimumab. Her response to the drugs was less than satisfactory and she had six readmissions in nine months for exacerbations of lower limb PG.

Surgical intervention can worsen PG through pathergy [2, 19, 20]. Therefore, surgical intervention such as SSG should only be performed in conjunction with immunosuppression. Mild debridement of necrotic tissue may prevent bacterial infections. All our patients who underwent SSG or debridement while on immunosuppressive therapy did not have any documented evidence of postoperative exacerbation of the skin disease.

### 4.5. Patient Outcomes

A literature search revealed little information on the prognosis of patients who were admitted for treatment of PG. Our study revealed that patients had lengthy hospital admissions (mean LOS: 47 days), high death (21.7%), and recurrence rates (39%). Patients who were admitted to hospital for treatment tended to have severe and aggressive PG. There are some factors which can affect the prognosis of PG. Reichrath et al. suggested that the type and severity of the associated systemic disease can affect the prognosis of PG [8]. Unresponsiveness of the associated disease to treatment resulted in a poorer prognosis. This is consistent with our findings in which 80% of the patients who died in our study had an associated systemic disease. Age is also a strong prognostic factor. The mean age of patients who died in our study was 78.8 years, 16 years older than the mean age of patients recruited for our study. In addition, the patients who died in subsequent readmissions were also old, with a mean age of 68 years at time of diagnosis of PG.

Our results also suggest a possible correlation between infected PG wounds and poorer prognosis which has never been reported in the literature. All the patients who died in our study had findings suggestive of infected lower limb PG and the cause of death was sepsis in 80% of the cases. We noted that patients with infected PG also had prolonged hospital stays. Infected PG requires urgent treatment with antibiotics and continuation of immunosuppressive medications. Reichrath et al. reported the use of topical treatments such as antiseptic or occlusive dressings in preventing wound infections [8]. However, none of these topical treatments have been used on our study patients.

Ulcerative variant of PG is more likely to be associated with poorer prognosis than other variants [3]. In our study, 80% of patients who died during initial admission had ulcerative PG. Corticosteroids remain the primary immunosuppressive treatment [7, 8]. Both monotherapy and combination therapy have similar efficacies in the treatment of aggressive PG [8]. Topical corticosteroid can be effective in treatment for small and superficial ulcers [8] and this is shown in the treatment of two of our patients with mild nonulcerative PG who received betamethasone dipropionate twice a day. Recurrence of PG requiring inpatient management was quite high (39%). Patients with lower limb PG are more likely to have recurrent PG requiring hospital admission. This highlights the importance of long term monitoring and follow-up in this group of patients.

### 4.6. Limitations

Due to the small sample size of this study, the results were not statistically analysed. PG is a rare disorder and recruiting large number of patients is extremely difficult. Other PG studies had similar number of cases to our study and all their results were not statistically significant [11–15]. Another limitation of our study is that not all our cases had wound biopsies. This raises the possibility of information bias occurring—the ulcers in patients who did not have wound biopsies may be caused by other conditions such as vasculitis and malignancy since PG is a diagnosis of exclusion. We felt that this possibility is quite low as the diagnoses of PG in all cases were made by dermatologists and vasculitic blood tests were negative.

## 5. Conclusion

The findings of our study suggest a poor outlook for patients with PG requiring hospital admission, with long hospital stays, high death, and recurrence rates. Factors possibly associated with poorer prognosis are age, ulcerative variant of PG, presence of associated systemic disease, high CRP levels, and clinical signs of wound infections. It is hence important to treat modifiable factors such as associated systemic diseases and wound infections. The presence of abnormally high CRP levels on admission and clinical features of infection are highly suggestive of infected PG and require a combination of intravenous broad-spectrum antibiotics and immunosuppression.

## Figures and Tables

**Table 1:** Demographics of 23 patients admitted with PG.

Demographics	Number of cases (%)
Age at diagnosis (years)	
30–44	4
45–59	4
60–74	10
75 and above	5
Mean age at diagnosis (range)	62.8 years (30–89)
Sex	
Male	7
Female	16
Associated disease	11
Treatment	
Topical	2
Systemic	21
Mean length of hospital stay (range)	47 days (5–243)
Outcome	
Alive after 1st admission	18
Dead at 1st admission	5
Recurrence	7

**Table 2:** Characteristics of patients who died during initial admission.

Age/sex	LOS (days)	Site	Type	Associated disease	Other comorbidities	Wound culture	CRP	Treatment	Cause of death
85 M	20	Lower limb	Ulcerative	Lung and prostate cancer	Atrial fibrillation, osteoporosis	*Pseudomonas *	65	Prednisolone, MMF	Sepsis
81 F	15	Lower limb	Vegetative	—	—	*Streptococcus*, *Enterococcus *	88	Prednisolone, minocycline	Sepsis
72 F	151	Lower limb	Ulcerative	Colon cancer	Aortic valve replacement, osteoarthritis	*Staphylococcus *	178	Prednisolone, dapsone, MMF, and doxycycline	Sepsis
72 F	38	Lower limb	Ulcerative	CREST	Osteoporosis, hypertension	*Staphylococcus *	111	Prednisolone	Multiorgan failure
83 M	29	Lower limb	Ulcerative	Monoclonal gammopathy	Psoriasis, gout, hypertension, and osteoporosis	*Pseudomonas*, *Staphylococcus *	52	Prednisolone, azathioprine	Sepsis

**Table 3:** Characteristics of patients who survived initial admission.

Age/sex	Site	Type	Associated disease	Other comorbidities	Treatment	Recurrence
67 M	Lower limb	Ulcerative	Ulcerative colitis	Stroke, hypertension, atrial fibrillation, and smoker	Prednisolone	Yes
45 M	Upper limb	Ulcerative	—	Hypertension, hypercholesterolaemia, depression, and obstructive sleep apnoea	Prednisolone, doxycycline	Yes
66 M	Peristomal	Vegetative	Crohn's disease	Ischaemic heart disease, hypertension, and peptic ulcer	Mometasone furoate ointment	Yes
30 M	Multiple	Bullous	Ankylosing spondylitis	—	Prednisolone, dapsone	Yes
70 F	Lower limb	Ulcerative	—	Diabetes type 2, hypertension, hypercholesterolaemia, ischaemic heart disease, congestive cardiac failure, and atrial fibrillation	Prednisolone, minocycline	Yes
33 F	Lower limb	Pustular	—	Obesity	Prednisolone, azathioprine, adalimumab, and MMF	Yes
53 F	Lower limb	Ulcerative	—	Obesity, osteoarthritis	Prednisolone, azathioprine	Yes
71 F	Lower limb	Ulcerative	—	Diabetes type 2, hypothyroidism, and peripheral vascular disease	Prednisolone	No
72 F	Lower limb	Ulcerative	—	Diabetes type 2, hypertension, and hypercholesterolaemia	Betamethasone dipropionate ointment	No
51 F	Multiple	Ulcerative	—	Diabetes type 2, chronic obstructive airways disease, hypercholesterolaemia, hypertension, and peripheral vascular disease	Prednisolone, MMF, and skin grafts	No
80 F	Peristomal	Ulcerative	Colon cancer	Hypertension, Guillain-Barre syndrome, diabetes type 2, and osteoarthritis	Prednisolone, minocycline	No
73 F	Peristomal	Ulcerative	Lung cancer	Hypertension, hypercholesterolaemia, and osteoarthritis	Prednisolone, doxycycline	No
41 F	Breast	Ulcerative	—	—	Prednisolone	No
60 F	Peristomal	Ulcerative	Colon cancer	Hypertension, ischaemic heart disease, and asthma	Prednisolone	No
51 M	Lower limb	Ulcerative	—	Diabetes type 2, ischaemic heart disease	Prednisolone	No
37 F	Breast	Ulcerative	—	—	Prednisolone	No
62 F	Breast	Ulcerative	—	Rheumatic heart disease, congestive cardiac failure, and atrial fibrillation	Prednisolone, doxycycline	No
89 F	Lower limb	Bullous	Essential thrombocytopenia	Diabetes type 2, hypertension, hypercholesterolaemia, osteoporosis, and peripheral vascular disease	Prednisolone, skin graft	No
